# A randomized controlled trial combining house screening and insecticide-treated nets reduces malaria transmission in northwestern Ethiopia

**DOI:** 10.1038/s41598-025-02943-7

**Published:** 2025-05-21

**Authors:** Aklilu K. Belay, Abebe Asale, Catherine L. Sole, Abdullahi A. Yusuf, Baldwyn Torto, Zewdu Abro, Menale Kassie, Clifford M. Mutero, David P. Tchouassi

**Affiliations:** 1 International Centre of Insect Physiology and Ecology, P.O. Box 30772-00100, Nairobi, Kenya; 2 International Centre of Insect Physiology and Ecology, P.O. Box 5689, Addis Ababa, Ethiopia; 3 Department of Zoology and Entomology, University of Pretoria, Private Bag X0028, Pretoria, South Africa; 4 School of Health Systems and Public Health, University of Pretoria, Private Bag X0028, Pretoria, South Africa

**Keywords:** Randomized controlled trial, Malaria, *Plasmodium* infection, Entomology, Epidemiology, Ecology, Medical research

## Abstract

House screening (HS) of doors, eaves, and windows using wire-mesh has demonstrated potential in the integrated vector management of malaria. However, limited epidemiological data are available to guide its implementation across different ecological settings. In a 16-month randomized controlled trial (follow-up period) conducted across three agroecological areas (dry mountain, plateau highland, and semi-arid) in Jabi Tehnan district, northwestern Ethiopia, treatment houses were equipped with HS combined with insecticide-treated nets (ITNs), while control houses received ITNs only. The intervention led to a significant 2.3-fold reduction in indoor malaria vector density, the primary entomologic outcome, largely influenced by *An. gambiae* s.l. mosquitoes. Fewer blood-fed mosquitoes were found in screened houses, indicating reduced human bites, which translated to six-fold decline in malaria prevalence (0.7%), the primary epidemiologic outcome, compared to control houses (4.3%). In contrast, *Plasmodium* sporozoite infection rates showed no differences between screened and control houses or agroecological zones, with *An. arabiensis* and *An. funestus* s.l. identified as the primary vectors. A modest protective effectiveness (22.6%) was observed, based on the estimated entomological inoculation rate of 0.24 and 0.31 infectious bites/person/night in screened and control houses, respectively, with no variation by agroecology. Despite the synergistic impact of HS with existing ITNs in reducing vector densities, human bite rates, and household malaria prevalence, sustained transmission persisted, partly due to the presence of highly competent vectors such as *An. funestus* s.l. which had an overall sporozoite rate of 68%. Future research should explore the interactions between vector behavioral adaptations, ecological and social factors contributing to residual transmission, even with seemingly effective control measures.

**Supplementary Information:**

The online version contains supplementary material available at 10.1038/s41598-025-02943-7.

## Introduction

Malaria is a deadly public health challenge, particularly in sub-Saharan Africa (SSA), which bears the brunt of the global malaria burden (94%); the disease contributes to a vicious cycle of poverty, underdevelopment, illness, and death^1^. According to the WHO, globally, nearly 233 million people are affected by malaria of which 580 000 die, each year, mostly children under the age of five years^1^. Conventional preventive strategies using insecticide-treated nets (ITNs) and indoor residual spraying (IRS) have been instrumental in controlling malaria worldwide, leading to a 60% reduction in the number of deaths, from 2000 to 2015 together^2^. However, since 2015, the progress in malaria control has stalled and gains made are now slowly being reversed, with increased disease prevalence which is evident in some malaria-endemic areas of SSA. Increasing insecticide resistance, changes in the behavior of primary vectors, the spread of cryptic vectors^3–5^ and invasive species (e.g., *Anopheles stephensi*)^6^, and climate change and variation are among the major threats to effective malaria control and barriers to elimination efforts^7^.

Additional vector control measures are needed to combat the persistent malaria problem, which can be implemented within the framework of integrated vector management (IVM), as highlighted in the WHO Global Technical Strategy for Malaria 2016–30^8^. House screening (HS) ─the use of fine wire-mesh screens to cover house doors, windows, and eaves, is among the promising approaches that can be adapted at the local level for effective and sustainable management of malaria^9,10^. Historically, house screening contributed to the eradication of malaria outside Africa^11,12^, yet it remains an under-utilized approach to control contemporary malaria^13^. The use of HS reduces man–vector contact by preventing people from the bites of malaria vectors that mainly occur indoors at night when in or before they go to bed. It represents a cheaper version or practical shortcut to house improvements to limit malaria infection^14^.

Recent studies have demonstrated the reduction in vector densities and *Plasmodium* infection rates, and the epidemiologic impact (e.g. decline in malaria parasite prevalence and anaemia) of HS on malaria^15–17^. These studies highlight the importance of HS as a supplementary tool for IVM, with added environmental value^11^. Indeed, untreated screens are environmentally friendly and do not require the use of chemicals that can induce resistance or behavioral changes in disease vectors. Additional benefits include less frequent replacements, ease of adoption, provision of protection to the entire household, and even during the day (when people are indoors but not in bed). Despite the proven effectiveness of HS against malaria, there is a lack of data to define eco-epidemiological settings where HS can be most effectively deployed. The WHO malaria prevention guidelines emphasize the need for further research on the impact of HS across different vector species, as variations in their behavioral divergence can significantly influence intervention outcomes^18^. Additionally, given the potential influence of local vector dynamics, variations in performance are expected if other local and environmental conditions affect the trial.

In this study, we hypothesize that HS can significantly reduce human-vector contact and mosquito entry, leading to decreased asymptomatic parasite prevalence and residual malaria transmission intensity among district inhabitants. By supplementing existing vector control measures, HS may prove effective across three agroecological zones with distinct malaria transmission dynamics and varying vector species compositions. To test this hypothesis, we conducted a randomized control trial (RCT) in the Jabi Tehnan district, northwestern Ethiopia, using empirical data to evaluate the entomologic and epidemiologic impacts on malaria prevalence and transmission. Ethiopia is among the five countries with the highest malaria burden globally, recording more than 1.3 million cases between 2021 and 2022^1^. The RCT compared house screening on doors, windows and eaves, combined with ITNs (intervention) to control houses that received only ITNs. The trial spanned three agroecological areas — dry mountain, plateau highland and semi-arid, to assess whether the effectiveness of HS varies by agroecology. Ultimately, our study aimed to evaluate the role of HS in mitigating residual malaria transmission in the district and to provide insights into its potential contribution to strengthening existing vector control strategies.

## Methodology

### Study area

**Fig. 1:**
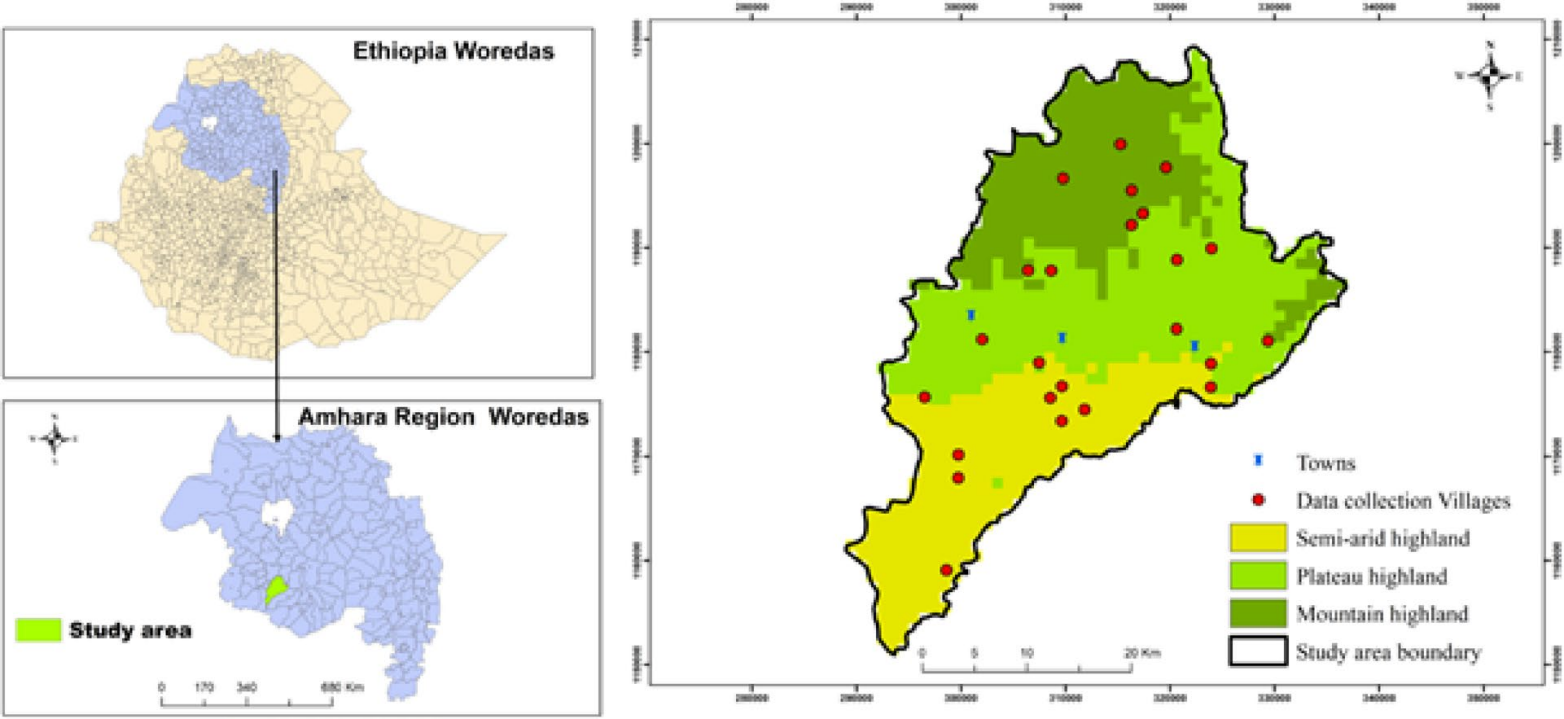
Map of the study area in Jabi Tehnan district, Amhara Region Ethiopia, showing the three major zones (Semi-arid, Plateau and Dry mountain highlands) and the villages (red dots) where sampling was conducted between July 2020 and October 2021.

The study was carried out in the Jabi Tehnan district (coordinates: 37.074° − 37.508°E; 10.405° − 10.945°N), situated within the West Gojjam Zone, Amhara Regional State of Northwestern Ethiopia (Fig. 1). The altitude of this subtropical dry area (semi-arid) is between 1300 and 2330 m above sea level (asl), and its topography is largely composed of plain (65%) and mountainous (15%) highlands. Mean daily temperatures range from 15 to 20 °C, and a long rainfall season of about three months (mid-June to mid-September), with an annual precipitation of 1356–1720 mm^19^. There are 41 villages in the district, with an estimated human population of over 228,351, distributed in 45,827 rural and 7,927 urban households^20^. The primary economic activity of the local population in the district is small-scale mixed farming, comprised of crop and livestock production^9,19^. Malaria transmission is bimodal- the peak season occurs after the heavy rains (June to September) and harvest period (Autumn), and it lasts between September and December; the second peak occurs during the short rainy season (Spring), between April to June^21^. There are about six streams that flow from north to south, dividing the district longitudinally at different distances from east to west. The plateau highland and semi-arid areas have partly irrigated arable land during the dry season which fuels the malaria incidence and is sustained throughout the dry months of January and February^9^. The district is divided into three zones, the major ones being semi-arid, plateau highlands, and dry mountain highlands (Fig. 1), classified using QGIS Geographic Information System (V3.30.0) software with a 30-meter resolution digital elevation model which was obtained from the United States Geological Survey.

### Study design

This interventional study which was conducted in Jabi-Tehnan district, Northwestern Ethiopia, was part of the larger project work for a Randomized Controlled Trial (RCT) on the combined impact of ITNs (DuraNet^®^ impregnated with the pyrethroid alpha-cypermethrin, Shobikaa Impex Pvt Ltd., Karur, Tamil Nadu 639,006, India), house screening, and pull-push technology for improved malaria control and livelihoods in rural Ethiopia^9,22^. The trial registration number Trial ID = 11,101, has previously been documented at the Pan African Clinical Trials Registry, PACTR202006878245287 on 24/06/2020^9^. The reconnaissance assessment found that 50% of households had at least one child aged ≤ 14 years in each treatment^9,22^.

We first prepared a list of 246 households for this study which included households that were similar, typically with mud-plastered walls, doors and windows made of furnished wood or metal sheet, and corrugated iron roofs. Then, we randomly selected 123 homes to receive ITNs plus screened their houses with fine wire mesh (doors, eaves, and windows). The remaining 123 households were assigned to the control group and received ITNs only. Notably, in this study, all the selected households received ITNs. The randomization study also considered differences in agroecological zones: dry mountain, plateau highland, and semi-arid areas. To enhance the randomization process, we stratified households by agroecological zone (dry mountain, plateau highland, and semi-arid) to ensure balanced allocation of intervention and control groups. Within each zone, households were further randomized to receive either ITNs plus house screening (*n* = 123) or ITNs only (*n* = 123). Given the potential influence of household characteristics on malaria risk, we ensured that selected homes were similar in structure (mud-plastered walls, wooden or metal doors, and corrugated iron roofs).

Malaria prevalence was estimated by employing active case detection during the following malaria transmission season post-interventions from asymptomatic house occupants. We conducted baseline epidemiological assessment before the intervention as presented in Asale et al.^23^. Most respondents (> 60%) kept cattle in a separate area from humans dwellings, whereas others shared it with cattle^9^.

### Data collection

The Centers for Disease Control and Prevention (CDC) miniature light traps (Model 512, John W. Hock, Gainesville, FL, USA), were used to collect indoor host-seeking mosquitoes bi-monthly, targeting two sampling rounds per season (autumn, dry, short and long rainy seasons) from July 2020 to October 2021, for a total of 8 rounds (sessions). Mosquito collection was performed for one night per household per sampling session. Each sampling session included control and intervention households in each of the three agroecological zones, providing comprehensive ecological coverage. Efforts were made to equilibrate the number of households in the control and intervention for trap setting. In each house, traps were positioned close to the sleeping quarters, approximately one meter above the floor. During each trapping session, sets of randomly selected residences were targeted to reduce the bias influenced by external factors. These included environmental conditions and human activities that might be unique to certain houses. Epidemiological assessments for *Plasmodium* infection were performed ten-months post-intervention using Rapid Diagnostic Tests (RDTs; The CareStart™ Malaria HRP2/pLDH COMBO Test, Access Bio Inc., USA) on selected asymptomatic household members, including children, school-aged individuals, and adults. Household member selection followed a predefined approach primarily focused on children, and if unavailable, a school-age or adult family member from the selected household was included.

The intervention was assessed over a 16-month follow-up period after the construction of house screening (HS). The primary entomologic outcome variable was indoor anopheline mosquito density. Secondary outcome variables including *Plasmodium* infection rates were used to estimate the entomological inoculation rates (EIRs), as a measure of the protective effectiveness of the HS. Other entomologic variables compared between screened and control houses included mosquito richness and host feeding patterns. *Plasmodium* parasite prevalence, as the primary epidemiologic outcome data, was estimated at the household across different age groups using RDTs.

### Mosquito identification and processing

Established taxonomic keys were used to identify mosquitoes microscopically (Coetzee, 2020). They were then grouped as unfed, blood fed, half gravid, or gravid^24^. The head/thorax of individual adult female mosquitoes were extracted using Genomic DNA (gDNA) based on the Genomic DNA ISOLATE II Kit (Bioline, Meridian Bioscience, Germany). We used PCR (Polymerase Chain Reaction) to detect *Plasmodium* DNA in the individual mosquito samples, as described previously by Belay et al.^3^. This encompassed multiple gene regions for *Plasmodium* infection screening, including markers targeting mitochondrial and ribosomal regions, as well as single copy gene glutathione reductase^25^. Positive mosquitoes were scored as positive for sporozoites. Further confirmation of a representative detected isolates was ensured by Sanger sequencing. We identified the sibling species in the *An. gambiae* complex using PCR^26^. We then processed the DNA which was extracted from the abdomen of individually engorged anophelines by PCR and sequenced them to identify vertebrate blood meal sources^3,27^.

### Data analysis

Female anopheline per trap was counted (i.e., mosquito densities). Densities for total anopheline and the predominant species, *An. gambiae* s.l. and *An. funestus* s.l. were analysed separately using Generalized linear mixed models (GLMMs), with negative binomial distribution. Agroecological areas, season (autumn, dry, short and long rainy seasons) and treatment houses (intervention vs. control) were included in the model as fixed effects and collection months (i.e. collection rounds) as random effect. The effect of the intervention on the captures of blood-fed mosquitoes were similarly compared using GLMM with negative binomial distribution. GLMMs were implemented with the *lme* package. We computed the pair-wise comparisons of mean densities between the agroecological areas and seasons using the package *emmeans* with an adjusted *p*-value based on Tukey test^28^. Further, we run a logistic binomial regression model to determine whether intervention effectiveness affected malaria prevalence by age group and agroecology. A similar model was performed to investigate the effect of the intervention on *Anopheles Plasmodium* infection prevalence; treatment and agroecology were included as explanatory variables with quasibinomial error distribution to adjust for overdispersion of the data. Bovine blood index (BBI) and human blood index (HBI) were estimated as the number of positive specimens of the total number analysed for a chosen species. The EIR (total sporozoite-positive mosquitoes by PCR/total samples screened) × (total anopheline catch/total CDC trapping efforts) was estimated as described previously^29^. All statistical analyses were carried out in R v. 4.2.1^28^ and results considered significant at *p* ≤ 0.05.

## Results

### House screening suppresses indoor *Anopheles* density and species richness

**Fig. 2:**
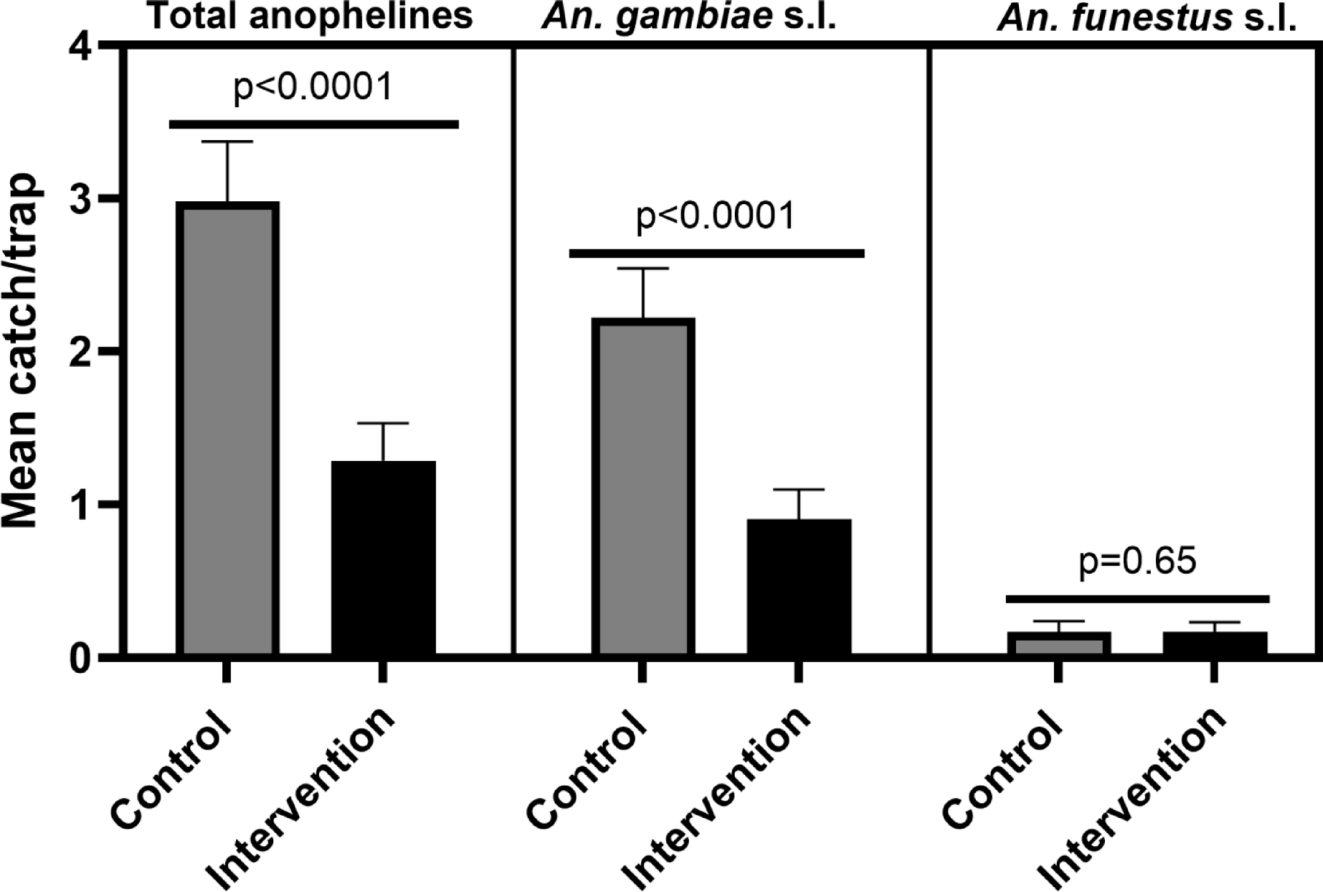
Impact of house screening on cumulative indoor anopheline densities in control and screened houses by agroecology in Jabi Tehnan District, Northwestern Ethiopia.

A total of 1,672 anopheline mosquitoes were collected throughout the 16-month follow-up survey, comprising 504 and 1,168 anophelines in screened and control households, respectively. The predominant mosquito species found in the traps was *Anopheles gambiae* s.l. (*n* = 1,224, 73.2%), out of 21 species collected, followed by *An. funestus* s.l. (*n* = 130, 7.8%). There was a two-fold significant reduction in indoor densities of total anophelines in screened relative to control houses (z =−5.95, *p* < 0.0001). A similar trend was observed for *An. gambiae* s.l. The intervention had no impact on the densities of *An. funestus* s.l., which was recorded in low numbers (Table 1; Fig. 2). There was no significant interaction between agroecology and intervention in influencing the densities of total anophelines (F value = 2.60, *p* > 0.05), *An. gambiae* s.l. (F value = 2.25, *p* > 0.05) and *An. funestus* s.l. (F value = 0.23, *p* > 0.05). This suggests that agroecology had no measurable impact on HS on mosquito densities. However, there was a discernible decline in the anopheline species richness, with only 13 species identified in treatment houses, and 19 species identified from the traps in the control houses (Supplementary Fig. S1 online).

**Fig. 3:**
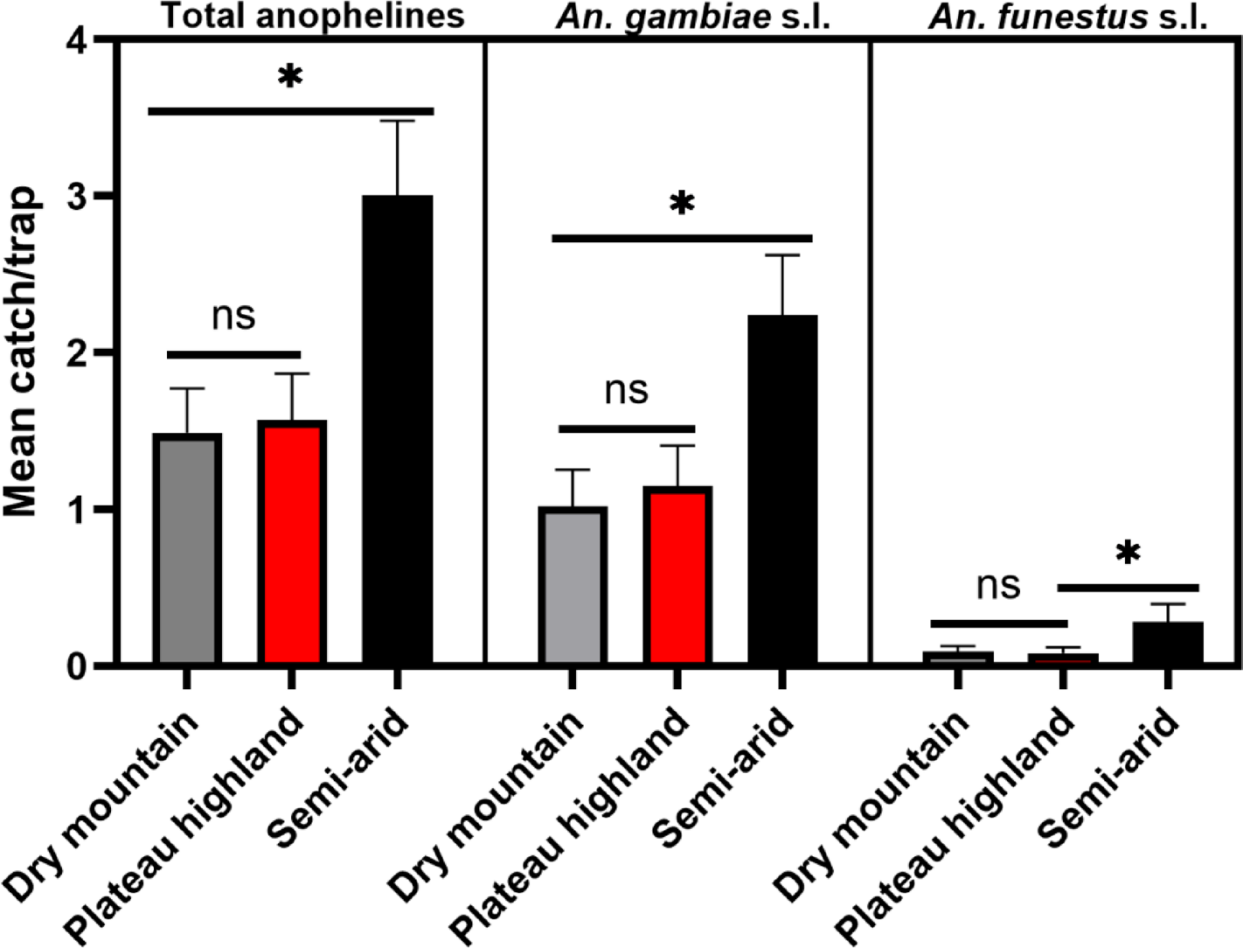
Agroecological differences in anopheline indoor densities in Jabi Tehnan District, Northwestern Ethiopia.

The densities of total anophelines, *An. gambiae* s.l. and *An. funestus* s.l. varied by season and agroecology (Table 1; Fig. 3). Total anopheline densities were significantly higher in the semi-arid (3 mosquitoes/trap) than in the dry mountain (1.5 mosquito/trap) (*p* = 0.04) and plateau highlands (1.9 mosquito/trap) (*p* = 0.02), with no significant difference between dry mountain and plateau highland (*p* = 0.99). A similar trend was mirrored by *An. gambiae* s.l. The occurrence of *An. funestus* s.l. was generally low, with variations in capture rates influenced by both season and agroecology. Seasonally, total anopheline abundance differed significantly between autumn and the long rains (*p* = 0.02) and short rains (*p* < 0.0001) but not the dry season (*p* = 0.99). Significant differences were also observed between the dry season and the long rains (*p* = 0.045) as well as the short rains (*p* = 0.0005), though no difference was found between the long and short rain seasons (*p* = 0.75). The abundance of *An. funestus* s.l. varied seasonally, with significant differences between autumn and the short rains (*p* < 0.0001) and between the long and short rain seasons (*p* = 0.003).

**Table 1:** Effect of house screening on anopheline vector densities in Jabi Tehnan district, Northwestern Ethiopia; models represent GLMMs with negative binomial distribution.

	Total anopheline abundance (df = 9, 775)			*Anopheles gambiae* s.l. (df = 9, 775)			*Anopheles funestus* s.l. (df = 9, 775)		
Factors	β estimate ± SE	Z value	*P*	β estimate ± SE	Z value	*P*	β estimate ± SE	Z value	*p*
Season: dry	−0.02 ± 0.27	−0.06	0.95	0.12 ± 0.39	0.316	0.75	−3.17 ± 1.081e + 06	0	1
: long rain	−0.82 ± 0.27	−2.97	0.003	−1.25 ± 0.44	−2.84	0.004	−1.00 ± 0.52	−1.93	0.053
: short rain	−1.08 ± 0.22	−4.87	< 0.0001	−1.22 ± 0.34	−3.54	0.0004	−3.75 ± 0.72	−5.21	1.92E-07
Agroecology: plateau highland	0.01 ± 0.24	0.03	0.98	0.06 ± 0.27	0.22	0.82	−0.04 ± 0.64	−0.06	0.95
: semi-arid	0.57 ± 0.24	2.41	0.02	0.55 ± 0.27	2.02	0.04	1.21 ± 0.58	2.08	0.04
Treatment: Intervention	−1.06 ± 0.18	−5.95	2.67E-09	−1.20 ± 0.20	−5.94	2.00E-09	−0.20 ± 0.44	−0.46	0.65

Autumn, dry mountain and control are used as reference categories for analysis of season, agroecology and treatment effects, respectively. df, degree of freedom; SE, standard error of the mean.

### House screening impacts blood-feeding rates and host range of *Anopheles* mosquitoes

The mosquito captures recorded 339 blood-fed anophelines, representing 20.3% (339/1672) of total captures. Of these, 83.5% (*n* = 283) were from the control houses, with 16.5% (*n* = 56) recorded from the screened houses. PCR analysis revealed weak or no band on the gel for 35 samples which could not be processed further. On the other hand, 253 (74.6%) engorged *Anopheles* were successfully genotyped for host blood DNA out of the remaining 304 samples. Of the sequenced samples, 211 (83.4%) were from the control houses and 42 (16.6%) from the intervention houses. The HS intervention significantly reduced blood feeding based on number of blood-fed mosquito captures compared to those in control traps (56 vs. 283; z-value= −8.21, *p* < 0.0001). Cattle emerged as the primary blood source for anophelines, with BBI of 73.5% (186/253), followed by the human host (HHI = 12.6%, 32/253). In the control houses, the HBI and BBI were 10.9% (23/211) and 76.3% (161/211), respectively. Surprisingly, screened houses had higher rates of HBI and BBI of 19.0% and 59.5%, respectively, than the control.

*Anopheles gambiae* s.l. had the largest proportion of blood-fed individuals (*n* = 256, 76%) among all species. The number of blood-fed *An. gambiae* s.l. was five times lower in the intervention households (*n* = 41; 16.0%) compared to control households (*n* = 215;83.9%). Most of the engorged *An. gambiae* s.l. (44%) were caught in the semi-arid (*n* = 113), whereas 28% were found each in the dry mountain (*n* = 73) and plateau highland (*n* = 70) zones. Regardless of the HS status, the HBI was higher in the semi-arid and plateau highlands, than in the dry mountain households in both groups. However, proportionally, human biting was higher in the screened houses (HBI = 19.1%, 8/42) than in control houses (HBI = 10.9%, 23/211). On the other hand, the BBI in the intervention, 59.5% (25/42) was lower than in the control houses 76.3% (161/211).

**Fig. 4:**
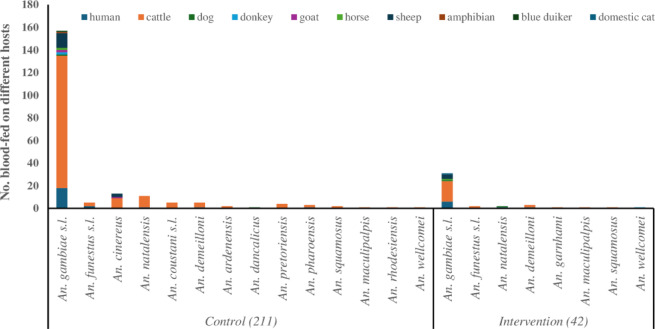
Impact of house screening on blood feeding proportions and host range among *Anopheles* mosquitoes in Jabi Tehnan District, Northwestern Ethiopia.

The intervention reduced the species richness of blood-fed anopheline, with only 8 species observed in intervention households compared to 19 species in the control households (Fig. 4).

### *Anopheles* infection prevalence and entomological inoculation rate

The detection of *Plasmodium* sporozoite was performed on a subset of 428 mosquito samples, comprising 253 blood-engorged and 175 gravid specimens. The control houses had 362 (engorged + gravid), whereas the intervention houses had 66 (engorged + gravid). A total of 11.2% (*n* = 48) of these mosquitoes tested positive for *Plasmodium* sporozoite infection. Of these, the intervention houses had a higher parasite rate (18.2%; 12/66) than the control houses (9.9%; 36/362). Among all examined anopheline species, 93.8% of infections were attributed to the two predominant species: *An. gambiae* s.l. (*n* = 27, all *An. arabiensis*) and *An. funestus* s.l. (*n* = 18). The remaining infections (*n* = 3) were each found in a single specimen of *An. pharoensis*,* An. cinereus*, and *An. natalensis* (Table 2). The intervention did not significantly reduce the mosquitoes infected with sporozoites (F-test = 3.3, df = 1, *p* = 0.09), and there was no significant variation induced by agroecological area (F-test = 1.74, df = 2, *p* = 0.18).

**Table 2:** Impact of house screening intervention on malaria parasite infection of anophelines across agroecology in Jabi Tehnan district, Northwestern Ethiopia.

Agroecology	Treatment	*An. gambiae* s.l.	*An. funestus* s.l.	*An. pharoensis*	*An. cinereus*	*An. natalensis*	Total
Dry mountain	Control	6 (77)	6 (6)	1(4)	1(17)	0(2)	14(106)
	Intervention	5(15)	3(4)	0(0)	0(0)	0(1)	8(20)
Plateau highland	Control	8(90)	3(4)	0(0)	0(1)	0(0)	11(95)
	Intervention	0 (12)	0(0)	0(0)	0(3)	0(1)	0(16)
Semi-arid	Control	7(108)*	4(7)	0(0)	0(0)	0(11)	11(126)
	Intervention	1(17)	2(3)	0(0)	0(6)	1(4)	4(24)
Total	Control	21(275)	13(17)	1(4)	1(18)	0(13)	36(362)
	Intervention	6(44)	5(7)	0	0(9)	1(6)	12(66)

*=Two of the positive samples tested positive for *Plasmodium vivax*; all other samples tested positive for *P. falciparum*. The numbers in parenthesis indicate the number of engorged/gravid specimens analyzed for each species.

The most frequent parasite detected was *Plasmodium falciparum*, infecting 95.8% (*n* = 46) of mosquitoes, with only two mosquitoes infected with *P. vivax*. The entomological inoculation rate (EIR) was 0.31 infectious bites per person per night (ib/p/night) in control houses and 0.24 ib/p/night in intervention houses, representing a 22.6% reduction in infectious bites (0.24/0.31 = 77.4% protection) (Table 3). The control and intervention EIRs of 0.29 and 0.31 ib/p/night, respectively by different agroecological zones were not significantly different between dry mountain and semi-arid environments. In contrast, the intervention provided complete protection in the plateau highland agroecology, where EIR was 0.32 ib/p/night in the control houses but zero in intervention houses.

**Table 3:** Impact of house screening on estimated EIR for the main malaria vectors across agroecology (July 2020-October 2021) in Jabi Tehnan district, Northwestern Ethiopia.

Agroecology	Treatment	*N*	Total Catch	SR	(ib/*p*/*n*)	*An. arabiensis*	*An. funestus* s.l.
Dry mountain	Control	100	221	11.8% (14/119)	0.26	0.12	0.09
	Intervention	100	76	33.3% (8/24)	0.25	0.18	0.07
	Total	200	297	15.4% (22/143)	0.23	0.12	0.08
Plateau_highland	Control	132	382	11% (11/100)	0.32	0.20	0.08
	Intervention	132	110	0% (0/14)	0.00	0.00	0.00
	Total	264	492	9.6% (11/114)	0.18	0.11	0.07
Semi-arid	Control	148	565	7.7% (11/143)	0.29	0.19	0.19
	Intervention	148	318	14.3% (4/28)	0.31	0.09	0.21
	Total	296	883	8.8% (15/171)	0.26	0.14	0.20
Grand Total	Control	380	1168	9.9% (36/362)	0.31	0.17	0.14
	Intervention	380	504	18.2% (12/66)	0.24	0.13	0.12
Grand Total		760	1672	11.2% (48/428)	0.25	0.14	0.13

N = no. of replicate traps; SR (no. positive/no. tested) = *Plasmodium* sporozoite rate; ib/p/n = infectious bite/person/night.

### Epidemiological assessment of HS intervention

Epidemiological assessment was conducted on a total of 1,490 individuals from control (*n* = 1053) and intervention (*n* = 437) households using RDTs. The overall malaria prevalence in control households was 4.3% (45/1053), 58.3% *P. falciparum* (*n* = 27/45), 39.5% *P. vivax* (*n* = 17/45), and 2.2% mixed infections (*n* = 1/45), and was significantly higher than that found in the intervention households (0.7%, 3/437; χ^2^ = 8.4, df = 1, *p <* 0.004). The prevalence rate for *P. falciparum* was 2.6% vs. 0.5% and 1.61% vs. 0.5% for *P. vivax* in the control and intervention houses, respectively.

The intervention significantly reduced (~ 6-fold) the malaria prevalence among household members (OR = 0.16;95% CI:0.04–0.46; *p* = 0.003). This suggests an 84% reduction in the odds of malaria in intervention households compared to control households. The intervention had a significant impact on individuals older than 14 years, with ~ 3-fold reduction in malaria risk (Table 4). This age group comprised 70.8% of the surveyed population (control = 720, intervention = 336). Gender was not a significant predictor of malaria risk (Table 4), and malaria prevalence did not vary by agroecology (Table 4).

**Table 4:** Binomial logistic regression analysis of intervention effects on asymptomatic malaria prevalence: evaluating odds ratios by gender, age, and agroecology.

Factors	OR (95% CI)	*p*-value
Treatment: Intervention	0.16 (0.04–0.46)	0.003*
Gender: Male	0.71 (0.39–1.33)	0.27
Age group: >14	0.56 (0.22–1.70)	0.25
: 5–14	1.43 (0.55–4.44)	0.49
Agroecology: Plateau highland	1.03 (0.50–2.22)	0.94
: Semi-arid	1.45 (0.63–3.41)	0.39

*= statistically highly significant compared to control reference, reference category of variables (gender = female, age group = < aged < 5, treatment = control houses, agroecology = dry mountain).

## Discussion

Our results demonstrate that HS, when combined with ITNs, has both entomological and epidemiological impacts on malaria transmission. Notably, the intervention resulted in a significant two-fold reduction in indoor densities of host-seeking anophelines, accompanied by a decline in anopheline species richness. Much of this reduction was driven by *An. arabiensis*, the primary malaria vector in Ethiopia^3,30,31^. The protective effectiveness of HS showed an overall 22.6% reduction in estimated EIR. Additionally, there was a notable decrease in the number of blood fed mosquitoes, indicating reduced biting activity. Collectively, these effects translated into a six-fold reduction in malaria prevalence in screened houses compared to control houses.

Previous studies have shown that HS is associated with a decrease in indoor malaria vector abundance and/or malaria transmission rates, as measured by EIR, in rural southeastern Zambia^32^, western Kenya^33^, and coastal Tanzania^12^. Furthermore, the positive impact of HS has been documented in reducing human infection prevalence^12,33^, malaria incidence^15^, and anaemia in children^16^. Our study provides additional evidence of the synergistic effect of HS with existing ITN-based control strategies, highlighting its potential to enhance malaria elimination efforts. Importantly, using insecticide-free screens, as in the current study, offers a sustainable malaria control strategy that is not susceptible to the physiological resistance observed in insecticide-based interventions such as ITNs and IRS.

Our findings suggest that the observed reduction in vector densities may reflect decreased mosquito-human contact or reduced biting rates. Few bites resulted in a low rate of engorged mosquitoes, which may explain the overall lower infection prevalence in humans. While this may provide strong support for HS as an effective household-level intervention^18^, it had only minimal effect on mosquito *Plasmodium* positivity rates. This indicates a non-linear relationship between human parasite prevalence and estimated EIR. Several factors may contribute to the observed lack of effect on parasite transmission. First, mosquito infectivity likely represents an indirect, community-level effect of HS, which may only be accurately assessed with broader intervention coverage^34^. Second, human or household factors could influence vector exposure risk. For instance, the higher mosquito parasite rate observed may be linked to the substantial proportion of human blood meals in both control and intervention houses, particularly if a small number of parasite reservoirs disproportionately drive human-to-mosquito transmission^34–36^. Additionally, both *An. arabiensis* and *An. funestus* s.l., the dominant vectors in our study are highly competent malaria transmitters^3,30,37^. Further research is needed to identify the human, environmental and mosquito-related factors contributing to residual transmission despite seemingly successful control measures. Understanding the interplay of these factors could help better define spatial risk and transmission hotspots, ultimately guiding more targeting malaria control strategies^38^.

The intervention significantly reduced overall malaria prevalence among household members, with a particularly pronounced impact in adults aged > 14 years. This difference may partially reflect a bias, although recent data suggests that this age group is now the most at risk for malaria. These results align with baseline studies, which found that adults (> 15 years) accounted for 50% malaria cases, followed by school-aged children (5–14 years) at 31%, and children under 5 at 10%^23^. The consistent reduction in malaria prevalence across age groups highlights the effectiveness of HS, which can be widely applied in malaria-endemic areas to provide significant protection to key population groups, including school-aged children and adults. Additionally, these findings suggest that HS offers broader protection for all household members indoors if used effectively.

We estimated the EIR as a measure of the protective effectiveness of the house-screening intervention^32^ using CDC light trap data. The CDC light trap is considered less prone to human bias, and EIR estimates from these traps offer an alternative to human landing collections^32,39^. Previous studies have shown a strong correlation between light traps and landing collection estimates^39^. The sporozoite rates were estimated from mosquito cohorts that were engorged, representing those that had previously bitten a host, including humans. Testing gravid and blood-fed cohorts increases the likelihood of detecting parasites, potentially introducing bias into the infection data. However, this cohort was consistently lower in intervention houses compared to their control counterparts. Since these mosquitoes are potentially older and more dangerous, the intervention may have indirectly impacted the longevity/survival of anophelines in our study. Further analyses incorporating age structure would be valuable for assessing future trials. Mosquito survival is a critical metric closely tied to the vectorial capacity of disease vectors^40^.

One of the limitations of our study is that intervention and control houses were not stratified by the presence of cattle near sleeping quarters, which may have influenced estimates of bovine blood-feeding rates. Additionally, light traps may not capture all anophelines with equal efficiency. The EIR was aggregated over the entire 16 month trapping period (post-intervention) due to the total number of captures and those analysed for infection presence, which limited the ability to explore seasonal variations. Passive prevalence data was collected after 8 months of intervention, but no clear relationship with entomologic data could be identified. Our analysis precluded accounting for potential household effect on the mosquito data. While we enforced a random design, a repeat of some households no more than twice occurred throughout the vector survey period. It is conceivable that this limited number of repeated measures per household likely provides insufficient data to reliably estimate household-level variance components. Accurate scoring for *Plasmodium* sporozoite detection could be affected by specific types of genomic DNA markers. To limit this potential issue, our data focused on analysis of head/thorax region and combining a suit of markers. Unequal trapping across agroecological zones was another unexpected issue, attributed to failed batteries whose data were excluded from the analysis. Overall, the data revealed low densities, consistent with previous reports in these ecological settings^3,41^.

## Conclusions

Our study demonstrates the benefits of HS of doors/windows/eaves, in reducing indoor host-seeking abundance of malaria vectors, dominated by *An. arabiensis*. This reduction reflects the proportion of mosquitoes engaged in blood feeding, indicating decreased biting activity, which ultimately leads to a decline in malaria prevalence. Agroecology had no effect on the performance of the intervention. The protective effectiveness of HS based on estimated EIR, was modest (22.6%), suggesting sustained transmission due to competent vectors such as *An. arabiensis* and *An. funestus* s.l. mosquitoes. Additional studies are needed to explore the interactions between vector behavioral adaptations and ecological and social factors that contribute to residual transmission, even with seemingly effective control measures.

## Electronic supplementary material

Below is the link to the electronic supplementary material.

Supplementary Material 1

## Data Availability

Data is provided within the manuscript or supplementary information files.

## Abbreviations

HS: House Screening
ITNs: Insecticide Treated Nets
CDC: Centre for Disease Control
EIR: Entomological Inoculation Rate
SSA: Sub-Saharan Africa
WHO: World Health Organization
IRS: Indoor Residual Spray
IVM: Integrated Vector Management
RCT: Randomized Control Trial
RDT: Rapid Diagnostic Test
PCR: Polymerase Chain Reaction
DNA: Deoxyribonucleic Acid
GLM: Generalised Linear Model
BBI: Bovine Blood Index
HBI: Human Blood Index

## Declarations

### Trial registration

The study protocol was registered online on May 28, 2020, with the Pan African Clinical Trials Registry (PACTR) at www.pactr.org. The registration number assigned to the protocol is PACTR202006878245287. The Trial’s methodology was also discussed by Asale et al.^9^.
