# Supplementary figures and images for: A randomized controlled trial combining house screening and insecticide-treated nets reduces malaria transmission in northwestern Ethiopia
Source: Sci Rep. 2025 May 21;15:17709. doi: 10.1038/s41598-025-02943-7 (PMC12095629; doi:10.1038/s41598-025-02943-7)

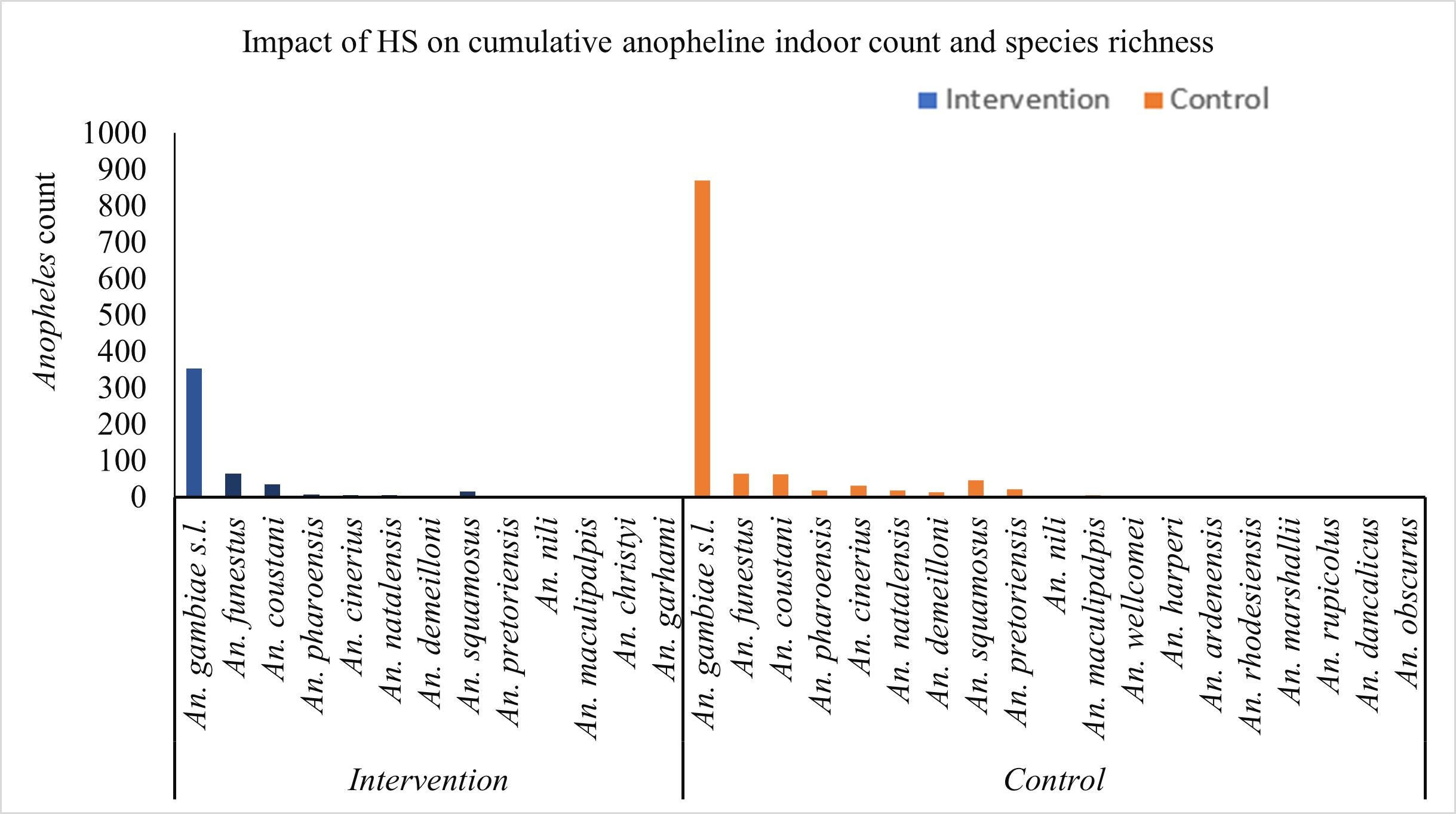

Supplement: Supplementary file 1 — Supplementary Material 1 [file 41598_2025_2943_MOESM1_ESM.jpg]
